# Identification of conserved and polymorphic STRs for personal genomes

**DOI:** 10.1186/1471-2164-15-S10-S3

**Published:** 2014-12-12

**Authors:** Chien-Ming Chen, Chi-Pong Sio, Yu-Lun Lu, Hao-Teng Chang, Chin-Hwa Hu, Tun-Wen Pai

**Affiliations:** 1 Department of Computer Science and Engineering, National Taiwan Ocean University, Keelung, Taiwan; 2 Graduate Institute of Molecular System Biomedicine, China Medical University, Taichung, Taiwan; 3 Institute of Biosciences and Biotechnology, National Taiwan Ocean University, Keelung, Taiwan

**Keywords:** Short tandem repeat, 1000 Genomes Project, CODIS, Next generation sequencing, genetic disease, orthologous gene, human-unique gene

## Abstract

**Background:**

Short tandem repeats (STRs) are abundant in human genomes. Numerous STRs have been shown to be associated with genetic diseases and gene regulatory functions, and have been selected as genetic markers for evolutionary and forensic analyses. High-throughput next generation sequencers have fostered new cutting-edge computing techniques for genome-scale analyses, and cross-genome comparisons have facilitated the efficient identification of polymorphic STR markers for various applications.

**Results:**

An automated and efficient system for detecting human polymorphic STRs at the genome scale is proposed in this study. Assembled contigs from next generation sequencing data were aligned and calibrated according to selected reference sequences. To verify identified polymorphic STRs, human genomes from the 1000 Genomes Project were employed for comprehensive analyses, and STR markers from the Combined DNA Index System (CODIS) and disease-related STR motifs were also applied as cases for evaluation. In addition, we analyzed STR variations for highly conserved homologous genes and human-unique genes. In total 477 polymorphic STRs were identified from 492 human-unique genes, among which 26 STRs were retrieved and clustered into three different groups for efficient comparison.

**Conclusions:**

We have developed an online system that efficiently identifies polymorphic STRs and provides novel distinguishable STR biomarkers for different levels of specificity. Candidate polymorphic STRs within a personal genome could be easily retrieved and compared to the constructed STR profile through query keywords, gene names, or assembled contigs.

## Background

Short tandem repeats (STRs), also known as short sequence repeats or microsatellites, are genome segments composed of short repeating sequences. The length of the fundamental repeat unit varies from one to six nucleotides [1]. STRs are highly abundant in many different organisms, and are distributed in both genic and intergenic regions[2]. Repeat structures expand or are deleted mainly due to replication slippage, which leads to highly polymorphic STR patterns among individuals [3]; these polymorphic features make STR motifs suitable genetic markers [4]. Several STR markers have been applied to individual/paternity identification and species/subspecies differentiation [5,6], while some STRs are involved in gene regulatory pathways. Abnormal expansion of such functional STRs located within coding regions frequently cause various types of disease [7,8]. Even when located within non-coding regions, STRs might also act as important functional regulatory elements [2,9]. Therefore, discoveries of polymorphic STRs among different sequenced samples might be helpful for detecting useful genetic markers, while findings of well-conserved STRs might lead to their identification as functional elements for gene regulation networks.

In traditional approaches, genomic STR markers have typically been discovered by analyzing DNA sequences through *in silico *methods and verified by PCR [10]. Various *in silico *tools are available for detecting both perfect and imperfect STRs within a single species [11,12]. In recent years, a revolutionary development in sequencing technology called next generation sequencing (NGS) has greatly impacted the growth and speed of genetic research. With relatively low costs and increased throughput, research at the genomic and transcriptomic levels has now become affordable and practical [13]. Traditional EST libraries applied to EST-STR discovery have been gradually replaced by NGS approaches, known as RNA-seq techniques, which provide extensive coverage at the whole-transcriptome level [14]. Recent publications have shown that NGS plays a low-cost and time-efficient role in polymorphic STR marker discovery, even without providing reference sequences [15]. The latest tools have also focused on STR marker discovery through NGS read analysis. For example, QDD is an open-source STR search tool package that provides a pipeline from raw NGS reads to STR identification and corresponding primer design [16]. Hoffman and Nichols also proposed a manual method for *in silico *STR marker screening [17]. Their experiments with Antarctic seals demonstrated the effectiveness of *in silico *STR marker discovery across individual NGS samples. The lobSTR program was developed by Gymrek *et.al*., who constructed a comprehensive survey of STR variations from NGS-derived personal genomes [18,19]. An automated method for detecting STR polymorphisms from NGS data reads could utilize the high throughput advantages of NGS without the influence of manually examined factors. In addition, we also developed a prototype system for detecting polymorphic STRs within human genomes based on the conception of an STR template profile [20]. However, due to our limited knowledge, there are no online web applications that allow users to compare personal genomes or specify genes for a comprehensive STR analysis. Therefore, we sought to develop an efficient identification system that is capable of detecting conserved and polymorphic STRs across different individual sequence reads. The proposed method could detect STR polymorphisms without curated procedures, and could be directly applied for the efficient identification of conserved and polymorphic STR markers and accelerate functional analysis of regulatory STR motifs.

## Results and discussion

We have performed a statistical analysis of the STR distributions in several datasets including chromosomal genes, combined DNA index system (CODIS) genes, disease-related genes, cross-species homologous genes, and genes that are unique to humans. The two major reasons for performing statistical analyses on different gene sets were: (1) to determine the most frequently appearing lengths of polymorphic/conserved STR patterns, and (2) the most frequently occurring regions of polymorphic STR motifs. To understand the extent of variation in the identified STRs, the distribution scale ranged from 1 to 84 bp. In addition, we selected the interval from 20 to 84 bp to analyze conserved degrees of identified STRs. Retrieved STRs from the different datasets are shown and discussed in the following sub-sections.

### CODIS marker analysis

CODIS is a collection of investigated and verified DNA markers provided by the U.S. Federal Bureau of Investigation (FBI) to criminal justice services. Thirteen STR markers (within ten defined genes and three intergenic segments) were examined in this program. From the verification results, our proposed system could successfully detect and list all 13 STR markers from 7 genomes, including six individual genomes from the 1000 Genomes Trio Project and one from the Ensembl reference genome. All retrieved STR markers are listed in Table 1, and it should be noted that both STR markers within the gene ***vWA ***(ENSG00000110799) defined by CODIS contain multiple short repeat patterns, and the adopted CGSSR program could successfully identify the three STR markers "ACAG", "AGAT", and "TCCA" for ***vWA***.

**Table 1:** Polymorphisms of CODIS STR markers for 7 different individuals.

CODIS marker	Repeat Pattern	Enbl.	NA12878 (CEU Child)	NA12891 (CEU Dad)	NA12892 (CEU Mom)	NA19238 (YRI Mom)	NA19239 (YRI Dad)	NA19240 (YRI Child)
**ENSG00000182578** **(*CSF1PO*)**	AGAT (Intron)	13.75	13.75	13.75	13.75	13.75	13.75	13.75

**ENSG00000011376** **(*D3S1358*)**	AGAT (Intron)	14.25	13.25	11.5	13.25	14.25	14.25	14.25

**ENSG00000249816** **(*D8S1179*)**	TATC (Intron)	13.75	13.75	13.75	13.5	13.75	13.5	13.75

**ENSG00000180176** **(*TH01*)**	AATG (Intron)	7.75	7.75	7.75	7.75	7.75	7.75	7.75

**ENSG00000171560** **(*FGA*)**	AAAG (Intron)	14.75	14.75	14.75	14.75	14.75	14.75	14.75

**ENSG00000171791** **(*D18s51*)**	GAAA (Intron)	15.5	15.5	15.5	15.5	15.25	15.25	15.25

**ENSG00000115705** **(*TPOX*)**	AATG (Intron)	8.5	8.5	8.5	8.5	8.5	8.5	8.5

**ENSG00000110799** **(*vWA*)**	ACAG (Intron)	5	3.75	4.75	3.75	5	5	4.75
	
	AGAT (Intron)	11.5	11.5	11.5	11.5	11.5	11.5	11.5
	
	TCCA (Intron)	12.5	12.5	12.5	12.5	10.25	11.5	10.25

**ENSG00000075213** **(*D7s820*)**	GATA (Intron)	10.75	10.75	10.75	10.75	10.75	12.25	10.75

**ENSG00000168367** **(*D16s539*)**	GATA (Intron)	11	11	11	11	11	11	11

***D5s818***	AGAT	11.75	11.75	11.75	11.75	11.75	11.75	11.75

***D13s317***	GATA	11	11	11	11	11	11	11

***D21s11***	TCTG	6.75	5.75	5.75	6.75	6.75	6.75	6.75

These genomes include the reference genome from Ensembl and 6 genomes (two families) from the 1000 Genomes Trio Project.

The first CEU family: NA12892, NA12891, and NA12878; the second YRI family: NA19238, NA19239, and NA19240. Enbl represents the genome from the Ensembl database; NA12878 represents the CEU daughter, NA12891 the CEU father, and NA12892 the CEU mother; NA19238 represents the YRI mother, NA19239 the YRI father, and NA19240 the YRI daughter. It should be noted that both STR markers of ***vWA ***were identified as 3 separate STRs in this table.

It was also observed that most polymorphic STRs within a family group agreed with inherited characteristics, i.e., the daughter's alleles were inherited from either one of her parents. Based on CODIS STR markers for comparing these two families from the Trio Project, the results show that 7 of 13 STR loci displaying identical repeat patterns/numbers among all selected individuals, and only one or two STR patterns possessing minor differences in length could be found between parents and daughter in both families. These results strongly suggest that distinguishability at the individual level in the post-NGS era would likely improve if more distinct STR markers were added to support CODIS.

### Disease gene analysis

To verify the accuracy and efficiency of the proposed system in detecting STR markers, we selected 13 well-known genes containing disease-related STRs. All identified STRs occurred in different genetic locations including protein coding regions, 5′ UTRs, 3′ UTRs and introns; large variations in repeat number might be causally related to serious genetic diseases according to previous medical reports. Table 2 lists all details including gene names, STR patterns and their genetic locations, expansion/deletion mechanisms, disease names, and references [8,9,21-30].

**Table 2:** A look-up table of genetic diseases, gene names, and corresponding STR patterns.

Gene	Repeat Pattern	Location	Normal Range (Repeat)	Disease- related Mutation	Related Disease	Reference
DMPK	CTG	3′UTR	5~37	Expansion	Myotonic Dystrophy Type 1	[21]

ATN1	CAG	Coding	7~25	Expansion	DRPLA	[22]

ATXN1	CAG	Coding	13~44	Expansion	Spinocerebellar Ataxia	[8]

EGFR	CA	Intron	14~22	Expansion	Breast Cancer	[23]

AR	CAG	Coding	10~36	Deletion	Hepatocellular Carcinoma	[24]

HTT	CAG	Coding	<28	Expansion	Huntington Disease	[25]

ATXN3	CAG	Coding	13~44	Expansion	Spinocerebellar Ataxia	[8]

FMR1	GCG	5′UTR	5~44	Expansion	Fragile × Syndrome	[26]

PABPN1	GCG	Coding	<10	Expansion	Oculopharyngeal Muscular Dystrophy	[27]

CACNA1A	CAG	Coding	4~16	Expansion	Spinocerebellar Ataxia	[8]

CALM1	AGC	5′UTR	20~45	Deletion	Prepro-calmodulin 1	[9]

ATXN10	AGAAT	Intron	10~22	Expansion	Spinocerebellar Ataxia	[28]

FXN	CTT	Intron	<12	Expansion	Friedreich Ataxia	[29,30]

Thirteen disease-related STR motifs located within coding, UTR, and intron regions were selected as test markers. The normal ranges of repeat number and variation types (deletion and expansion mechanisms) of STRs causing disease phenotypes are listed according to published references.

**Figure 1:**
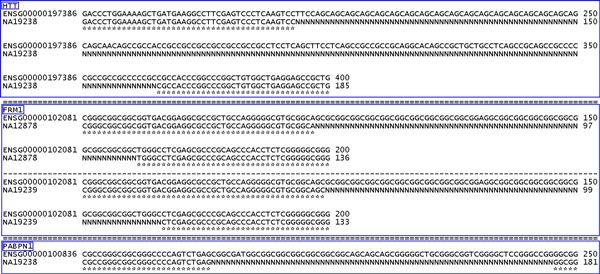
**Examples of undetected STRs for well-known genetic diseases**. STRs within three genes including HTT, FRM1, and PABPN1 for two individuals (NA19238 and NA12878) could not be identified. The three bounding boxes represent the aligned results for three different genes. The unsuccessfully detected STRs in both HTT and PABPN1 genes were only for the NA19238 genome, and failed STR detection in FRM1 occurred for both NA12878 and NA19238 genomes. Flanking sequences of these STRs were well-aligned and are indicated using "*" symbols. It can be observed that the target STR was not detected due to absence of consensus STR segments (shown with the character "N"). Missing nucleotides might be caused by NGS sequencing issues or errors created during applying the SAM tool.

The polymorphisms of disease-related STRs within all individuals were detected and compared, as shown in Table 3. The results show that 10 of 13 polymorphic STRs among all 7 individuals could be identified, and most repeat numbers fall within the normal range. However, three STR markers could not be retrieved from two individual samples (shown as 0* in the two individual IDs NA19238 and NA12878). The unsuccessful STR detection was mainly due to missing nucleotides in the consensus sequences. Figure 1 displays the undetected STR patterns by showing alignment results of the target STRs and corresponding flanking sequences between the reference profile and individual sequences. We observed that the individual consensus sequences were filled with the character "N" at the expected repeat locations; this might be due to NGS sequencing flaws or errors caused by the sequence alignment map (SAM) tool consensus output data. These examples of unsuccessful detection also indicate that the performance of the proposed system depends on the accuracy of NGS sequencing and reconstruction processes. In Table 3, none of the remaining successfully retrieved STRs showed abnormal patterns consistent with lethal diseases. Most of these regulatory STRs were identified in all individuals and were matched with family inheritance characteristics. Nevertheless, from the resulting tables we observed that two STR patterns located within the coding regions of the ***DMPK ***and ***AR ***genes were not consistent with heredity principles. This phenomenon might be a result of mixed sequencing data from heterozygous alleles. More recently developed assembly and reference mapping methods might be capable of distinguishing heterozygous alleles and overcoming such problems [31].

**Table 3:** Polymorphism of disease-related STR markers.

Gene Name	Enbl.	NA12878 (CEU Child)	NA12891 (CEU Dad)	NA12892 (CEU Mom)	NA19238 (YRI Mom)	NA19239 (YRI Dad)	NA19240 (YRI Child)
DMPK	20.666666	19	20.666666	17.666666	11	19.333334	20.666666

ATN1	15.666667	15.666667	15.333333	15.666667	12	15.666667	15.666667

ATXN1	14.666667	14.666667	14.666667	13	14.666667	14.666667	14.666667

	13.333333	13.333333	13.333333	13.333333	13	13.333333	13.333333

EGFR	22	22	22	22	22	21	21

AR	25.000001	23.666667	17.333334	24.333333	16.333334	18.666667	25.000001

HTT	19.666666	10.666667	11.333333	15.666667	0*	18	19.666666

ATXN3	8	8	8	8	8	8	8

FMR1	20.333333	0*	9.666667	2.666667	13.666667	0*	10.333333

PABPN1	7	3.666667	7	3	0*	7	7

CACNA1A	13.333333	13.333333	2	13.333333	13.333333	13.333333	13.333333

CALM1	7	8.333333	8.333333	8.333333	7	8.333333	7

ATXN10	14	14	14	14	14	14	14

FXN	6.666667	6.666667	6.666667	6.666667	6.666667	6.666667	6.666667

Enbl represents Ensembl; NA12878 represents the CEU child, NA12891 the CEU father, and NA12892 the CEU mother; NA19238 represents the YRI mother, NA19239 the YRI father, and NA19240 the YRI child. The * indicates that the specific STR could not be found due to sequencing errors or inconsistent reference mapping processes. This table shows that the STR pattern for the "FMR1" gene displayed obvious variations among different individuals.

### Polymorphic STR distributions for homologous and human-unique genes

To discover and distinguish important features of polymorphic STRs extracted from orthologous genes and human-unique genes within a human genome, we performed a statistical analysis of STR distributions from previously collected gene sets. In Table 4, the average occurrence rate of polymorphic STRs in all 225 homologous genes is 0.3216 (Polymorphic STRs/Mbp), which is less than the percentage of polymorphic STRs in 492 human-unique genes with a rate of 1.7020 (Polymorphic STRs/Mbp). This observation suggests that characteristics of STRs in homologous genes are highly conserved among various species. In other words, if homologous genes possessing highly variable STRs were conserved across species, this might lead to effects on important genetic functions. In addition, we compared the variation rates of CODIS STR markers, which were higher than the percentage of homologous genes but lower than the rates of human-unique genes. We speculate that the polymorphic STR patterns of these 492 human-unique genes should provide more identifiable STR markers than CODIS-selected genes, and might not be related to genetic functions for human beings or provide distinguishable features for different individuals.

**Table 4:** Occurrence rates of variation in STRs for 225 homologous genes, 10 CODIS genes (excluding 3 segments), and 492 human-unique genes.

Polymorphic STR Variation (bp)	TNpSTR	TLgene (Mbp)	TNpSTR/TLgene
≥ 1	357	1110.20	0.3216

(a) STR statistics for 225 homologous genes			

Polymorphic STR Variation (bp)	**TNpSTR**	**TLgene (Mbp)**	**TNpSTR/TLgene**

≥ 1	31	88.37	0.3508

(b) STR statistics for 10 CODIS genes			

Polymorphic STR Variation (bp)	**TNpSTR**	**TLgene (Mbp)**	**TNpSTR/TLgene**

≥ 1	477	280.26	1.7020

(c) STR statistics for 492 human-unique genes			

Human-unique genes exhibited the highest variation rate compared to CODIS and homologous genes. (TNpSTR represents the total number of polymorphic STRs and TLgene is the total length of selected genes in Mbp).

To observe the levels of STR marker variation within homologous genes, we calculated maximum deviation (*Max. Dev*.) and average deviation (*Avg. Dev*.) in base pairs. The definitions of *Max. Dev*. and *Avg. Dev*. are denoted in Eq (1) and Eq (2), respectively. *Max. Dev*. represents the largest number of repeat differences (in bp) of a specified STR within the identical genes from any two individuals, and *Avg. Dev*. is obtained by taking an average-of-length difference (in bp) between all corresponding STRs within the identical genes from all possible pair combinations among 7 individuals.

(1) $$Max.\;Dev.\left(a\right)=Max\left(\left|a_{i}\left(S_{k}\right)\right|\right)-Min\left(\left|a_{j}\left(S_{k}\right)\right|\right),\;i\neq j,\;S_{k}\in a_{i},\;a_{j}\;\text{for}\;\text{all}\;k$$

(2) $$Avg.Dev.=\frac{\sum_{i,\;i\neq j}^{7}\sum_{j}^{7}\left|\left|a_{i}\left(S_{k}\right)|-|a_{j}\left(S_{k}\right)\right|\right|}{\left(\begin{array}{c} 7\\ 2 \end{array}\right)}$$

where $\left|a_{i}\left(S_{k}\right)\right|$ is denoted as the repeat length of the STR $S_{k}$ within the selected "*a" *gene from the *i*^th ^individual, while $\left|a_{j}\left(S_{k}\right)\right|$ represents for the *j*^th ^individual.

We found that a total of 477 polymorphic STR patterns were detected in 492 human-unique genes, in which most of the patterns were located within "intron" regions. These results were similar to those for the CODIS STR markers. Additional file 1 lists the sorted STRs according to the *Avg. Dev*. and *Max. Dev*. To illustrate the differences in repeat length for each person, we selected two STR patterns with large differences among 7 individuals.

**Figure 2:**
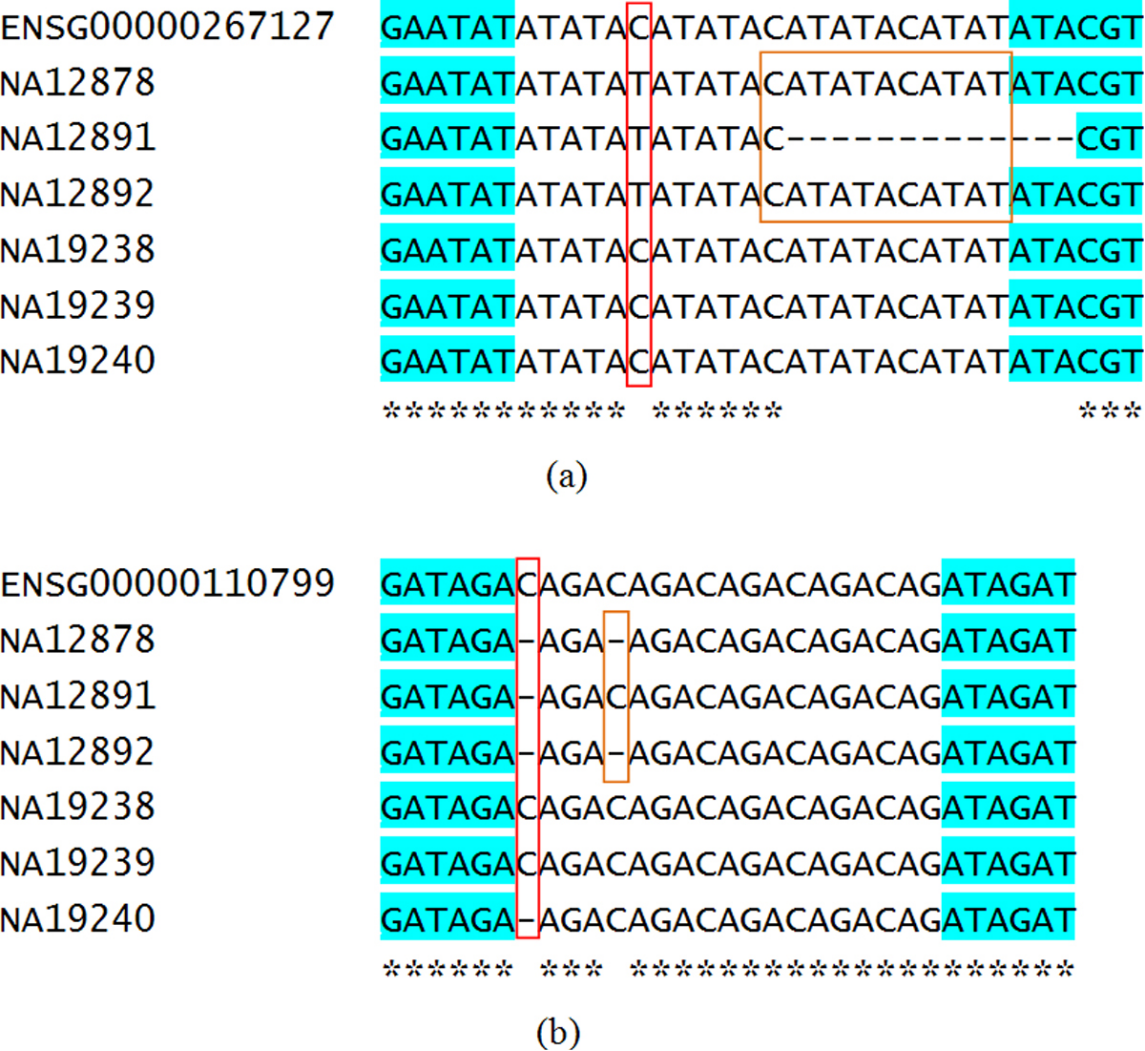
**Examples of different levels of polymorphic STRs**. The STRs were retrieved from ENSG00000267127 and ENSG00000110799 for all 7 human genomes. NA12878 represents the CEU child, NA12891 the CEU father, NA12892 the CEU mother, NA19238 the YRI mother, NA19239 the YRI father, and NA19240 the YRI child. (a) Aligned polymorphic STR patterns and flanking sequences for ENSG00000267127, which is contained in the human-unique gene set. Left red box shows the differences between each family, while the right orange box represents inheritance relationships within a family (identical STRs for both mom and daughter). (b) Aligned polymorphic STR patterns and flanking sequences for ENSG00000110799, which is contained within the CODIS gene set. Similar conditions for a previous example and the highlighted segments in blue background represent aligned flanking sequences.

In addition, we selected examples of polymorphic STR patterns with family inheritance relationships from all detected STRs. Two aligned results are shown in Figure 2. It is interesting to observe in Table 1 that the polymorphic STRs from CODIS gene sets were well-conserved in different families and individuals: a total of 8 defined STR biomarkers within 13 genes displayed exactly the same repeat pattern and length, and only one or two polymorphic STRs could be identified between any two individuals. Hence, how to increase distinguishability at different levels becomes an interesting challenge. Here we illustrate two STR examples in Figure 2 that showed variations in polymorphic STRs at different levels; such STR motifs could be further experimentally evaluated and applied to identify different individuals or groups.

According to the STR variations among 7 human genomes, we tried to define 3 distinct types for comparing polymorphic STRs. The first type of polymorphic STR represents a set of suitable STRs for distinguishing each individual, including the query sequences coming from members of the same family. The second type of polymorphic STR demonstrates a set of identified STR biomarkers obeying inheritance and could be applied to different groups. The last type of specific STR provides a set of suggested STRs that reveal characteristics that are identical for the Trio families but different from the other groups. A total of 26 specific STR biomarkers were defined from the identified 477 polymorphic STRs within human-unique genes. Additional file 2 lists all 26 relevant STR biomarkers, of which 17 markers appeared as a type of single nucleotide polymorphism (SNP). All of these 26 STRs demonstrate relatively high potential as distinguishable STR biomarkers at different levels.

### Polymorphic STR distributions in chromosomes

**Figure 3:**
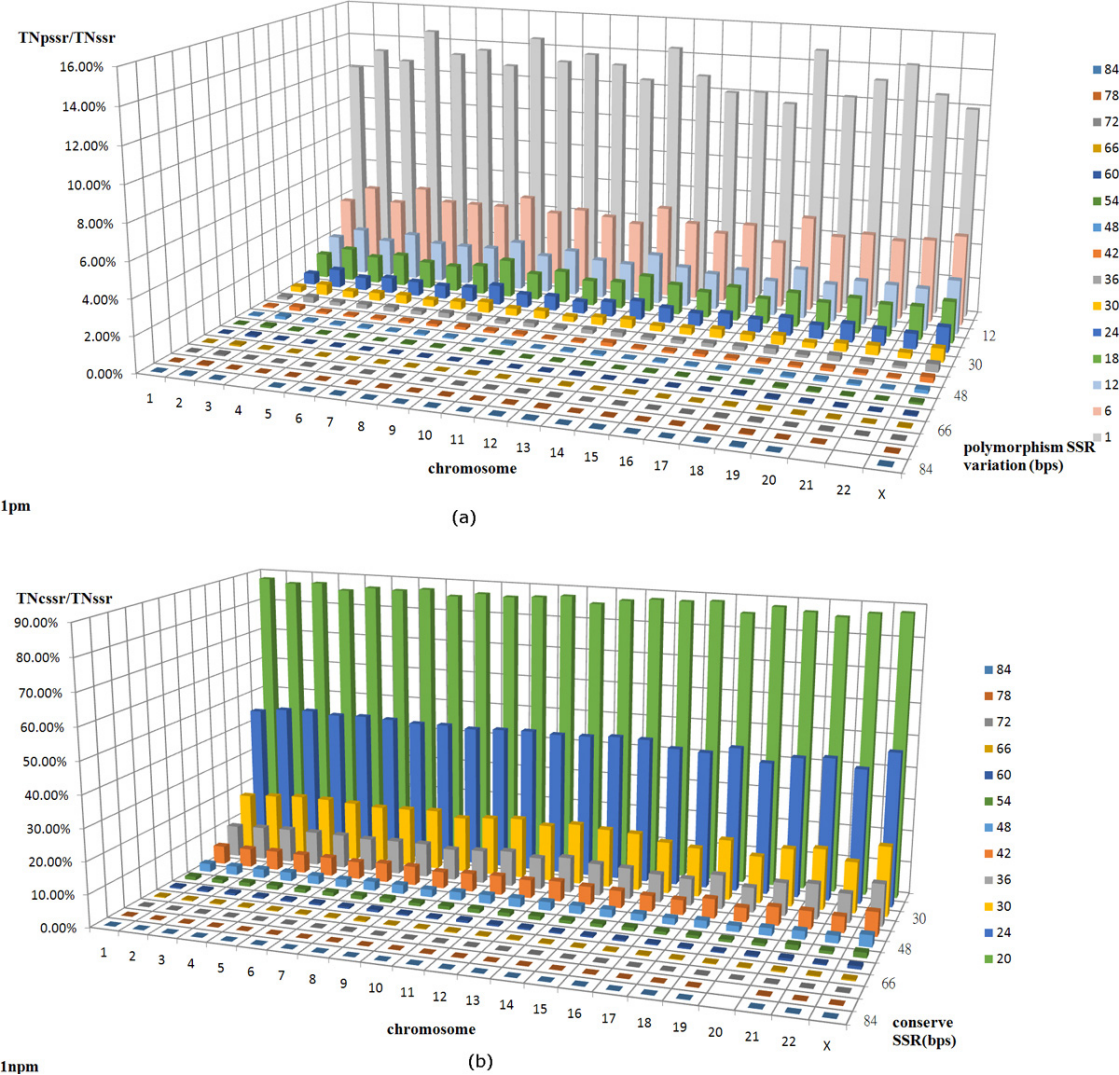
**Average percentages of polymorphic and conserved STRs for the selected 7 human genomes**. (a) Average polymorphic STR distribution in each chromosome. x-axis represents the chromosome number; y-axis represents the number of differences between varied STRs (bp) ranging from 20 to 84 bp; z-axis represents the accumulated percentage for each chromosome. The accumulated percentage of varied STRs was obtained by summing up average percentages from high to low variations for each chromosome. (b) Average conserved STR distribution in each chromosome. The x-axis represents the chromosome number; y-axis represents the number of conserved STRs (bp) ranging from 20 to 84 bp; z-axis represents the accumulated percentage for each chromosome. The accumulated percentage of conserved STRs was obtained by summing up average percentages from high to low conserved lengths for each chromosome

Polymorphic STRs identified from each chromosome were analyzed and compared for 7 individuals. The total number of genes is 56,852 in this study, of which 617 were not successfully detected due to serious sequence variations and/or query genes located at defined boundaries. Moreover, a successful rate of 98.91% was achieved for polymorphic STR analyses in this study. In addition, we did not consider STR motifs in the Y chromosome since it belongs exclusively to males. Hence, we only performed a statistical analysis of the STR distribution of both polymorphic and conserved STRs among all acquired genomes. Figures 3a and 3b show the distributions of polymorphic STRs and conserved STRs within each defined chromosome, respectively. In both figures, the *x*-axis represents the chromosome number, the *y*-axis represents the number of differences between varied STRs, and the *z*-axis denotes the accumulated percentages of polymorphic/conserved STRs in each selected chromosome. The highest bars in the last row (shown in light grey) in Figure 3a represent all accumulated percentages of polymorphic STRs for each chromosome, while the highest bars in the last row (shown in green) in Figure 3b represent all accumulated percentages of conserved STRs. The highest percentages of the corresponding bars from two figures should total 100% for each chromosome. For example, the percentage of polymorphic STRs in chromosome 1 is calculated by taking the total number of polymorphic STRs (TNpssr) within chromosome 1 divided by the total number of identified STRs (TNssr) within chromosome 1, and the average percentage of polymorphic STRs in the first chromosome for 7 human genomes is nearly 12.1%. Similarly, the average percentage of conserved STRs for chromosome 1 is obtained by taking the total number of conserved STRs (TNcssr) divided by the total number of identified STRs (TNssr). After taking the average from 7 human genomes, the percentage of conserved STRs is approximately 87.9% for chromosome 1. In other words, in chromosome 1, the total number of conserved STRs is more than 7-fold greater than the number of polymorphic STRs. It should be noted that the ratio of conserved STRs to polymorphic STRs is quite consistent for each chromosome, and the average fold change for all the different chromosomes is about 6.68.

Furthermore, we also evaluated the total length of STRs (TLSTR), total length of selected genes (TLgene), total number of genes (TNgene), total number of STRs (TNSTR), total number of polymorphic STRs (TNpSTR), density of polymorphic STRs, and occurrence ratio of polymorphic STRs in each chromosome. These data are summarized in Table 5, which shows that the highest density of polymorphic STRs was found on chromosomes 19 and 20 (with 0.921 and 0.780 polymorphic STRs per Mbp, respectively), and the lowest density was observed on chromosome 3 (with 0.375 polymorphic STRs per Mbp). It should be noted that the occurrence ratio of polymorphic STRs in each chromosome is distributed evenly within the range from 11.57% to 14.73%. However, these data show non-random associations between STRs and genes that could be observed from the distributions of the number of STRs, the gene number and gene length on each chromosome. For example, the total numbers of STRs retrieved from chromosomes 19 and 7 are 23255 and 24975, respectively, but the total numbers of genes are 2901 and 2792, respectively. As another example, the total numbers of STRs retrieved from chromosomes 19 and 8 are 23255 and 19247, but the total gene lengths are 3074.71 Mbp and 5590.48 Mbp, respectively. Greater gene lengths or higher numbers of genes do not imply the existence of repeat segments.

**Table 5:** Comprehensive STR statistics for all chromosomes, sorted by polymorphic STR density.

Chr	TLSTR (Mbp)	TLgene (Mbp)	TNgene	TNSTR	TNpSTR	TNpSTR/TLgene (Density, STRs /Mbp)	TNpSTR/TNSTR (Occurrence Ratio, %)
**19**	26.62	3074.71	2901	23255	2834	0.921	12.19

**20**	9.36	1668.80	1311	9836	1302	0.780	13.24

**X**	16.62	2983.31	2345	16769	2000	0.670	11.93

**22**	10.61	1674.64	1190	8716	1100	0.657	12.62

**13**	8.75	1977.35	1213	9002	1295	0.655	14.39

**9**	17.93	3533.43	2261	16386	2163	0.612	13.20

**10**	22.42	4403.78	2200	19231	2641	0.599	13.73

**21**	6.32	1226.41	711	5145	731	0.596	14.21

**16**	24.74	3858.90	2332	18462	2238	0.580	12.12

**6**	23.98	4997.39	2896	21135	2877	0.576	13.61

**1**	44.11	8295.52	5222	38217	4624	0.557	12.10

**17**	31.47	4813.97	2883	22240	2574	0.535	11.57

**8**	26.88	5590.48	2337	19247	2789	0.499	14.49

**7**	33.00	6499.22	2792	24975	3189	0.491	12.77

**5**	30.27	6396.45	2829	23459	3109	0.486	13.25

**4**	27.35	6111.19	2477	20358	2967	0.486	14.57

**12**	33.25	6107.63	2808	23543	2925	0.479	12.42

**14**	21.26	4147.19	2184	15323	1971	0.475	12.86

**2**	44.41	9299.27	3970	33220	4389	0.472	13.21

**15**	21.84	4292.70	2061	16459	1976	0.460	12.01

**11**	30.39	6155.13	3179	20975	2770	0.450	13.21

**18**	14.43	3121.40	1103	9448	1392	0.446	14.73

**3**	43.39	9234.03	3030	27336	3463	0.375	12.67

Chr = chromosome; TLSTR represents the total length of STRs in a query chromosome; TLgene is the total length of selected genes; TNgene is the total number of genes; TNSTR is the total number of STRs; TNpSTR is the total number of polymorphic STRs.

**Figure 4:**
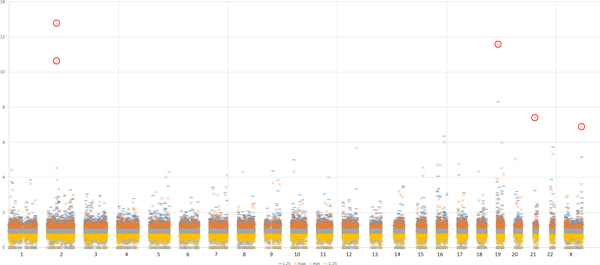
**A Manhattan-like scatter plot of all polymorphic STRs across the chromosomes of the human genome**. The *x*-axis represents genomic coordinates of the chromosomes in sequential order. The *y*-axis includes normalized upper and lower bounds of varied repeat number among 7 individuals (represent by "-" in two different colors). Upper/lower bound is calculated by multiplying +/-1.25 to upper/lower quartile of repeat number and normalized by dividing median value among 7 individuals. The *y*-axis also includes normalized maximum and minimum varied repeat numbers among 7 individuals (represented by symbol "x" in two different colors). At a threshold value of "6", 5 polymorphic STR patterns (circled symbols ) were considered as important biomarker candidates in this example.

Alternatively, highly variable STR patterns among 7 human genomes can be determined by assessing the extent of STR variations using a Manhattan-like scatter plot for all human chromosomes. The quality setting for all identified STR patterns is defined as 1.0 for this plot. Through the Manhattan plot (Figure 4), several polymorphic STR motifs exhibiting very high variation were readily apparent, and these extremely varied cases could be considered as the first choice for STR biomarker candidates. If a higher normalization threshold value for variation were assigned, fewer polymorphic STR biomarker candidates would be retrieved from the plot. For example, when the threshold value of variation was set to "6", the system replied with 5 important polymorphic STR candidates. These selected STR candidates are located within ENSG00000187627, ENSG00000233673, ENSG00000142453, ENSG00000154654, and ENSG00000029993 on chromosomes 2, 2, 19, 21, and X, respectively.

### The ISP online web system

We designed a comprehensive web-based system called ISP for efficiently identifying polymorphic STRs among different individuals. Several useful functions were designed for users to retrieve and verify all potentially important STR biomarkers and compare personal STRs to 7 published human genomes. Users can enter an Ensembl gene ID, gene descriptions, gene names, or any related keywords, and the system immediately responds with query results for the appropriate gene selection. Users can then select an interesting gene and a pop-up dialog for STR quality and STR variation settings is displayed on the web page. For real-time analysis, only two quality values of 1.0 and 0.9 are currently available, and variation degrees are automatically decided and unlocked for selection depending on the selected genes. A quality of 1.0 indicates that all identified STRs are perfect STRs, while a quality of 0.9 indicates that an identified STR contains less than 10% noise including mutations, insertions, and deletions. Variation degree is calculated as the true difference in base pairs between any two polymorphic STRs.

In the proposed system, users can provide customized sequences for STR polymorphism analysis. Once the query sequences are uploaded, the system will apply BLAST+ to align the query sequence against the reference human genome. Once the query sequence is successfully aligned to one of the collected human genes, the newly identified STRs within the query sequence are compared to all 7 human genomes for polymorphic STR analyses. The query results are exactly the same as described above. Here, the threshold for identity in BLAST+ was set at 99%. Such a relatively high threshold value avoids ambiguous situations caused by non-human sequences. Finally, the compared results are displayed via a tabulated interface and sent via email. For security reasons the URL was designed with embedded encryption.

**Figure 5:**
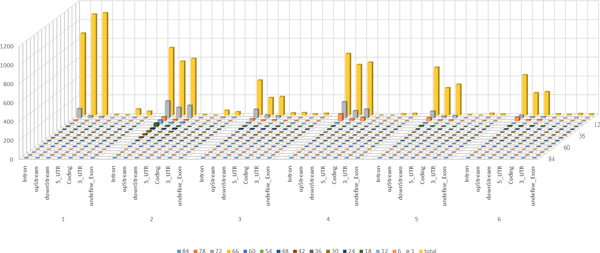
**An example of distribution profiles of polymorphic STRs on chromosome 6**. *x*-axis represents 6 different lengths of STRs (from mono- to hexa-nucleotide repeat units) located within 6 different genetic regions of polymorphic STRs; *y*-axis represents the accumulated number of base pairs for STR variations (from SNP to 84 bp variation); *z*-axis represents the total number of genes having polymorphic STRs.

The system also includes four test gene sets including disease-related genes, CODIS genes, homologous genes, and genes related to a GO term of GO:0001501. Corresponding statistical reports stored in Microsoft Excel files are provided in the developed system. For online queries for interesting genes, users can click on the folder "ISP Datasets", and four different gene sets and their corresponding identified polymorphic STRs are available for each individual gene. To comprehensively analyze polymorphic STRs for all human genes, the folder "Chromosome Statistics" provides 23 Excel files, each of which contains the total number of STRs, total number of polymorphic STRs, total length of selected genes, total length of STRs, percentages of exact genetic locations of all detected STRs, percentages of different variation degrees for all polymorphic STRs, and two different degrees of STR quality (perfect STR and imperfect STR with less than 10% noise content). All these statistics can be downloaded directly from the interface. One example of the polymorphic STR distributions on chromosome 6 with perfect STR quality settings is shown in Figure 5. When comparing the yellow bars in the last row, the group percentage of polymorphisms of mono-nucleotide STR motifs appears with the highest gene number, while the tri-nucleotide STR motifs comprise the lowest percentage of genes. Polymorphic STRs located within the coding regions (the fifth position in each distinct fundamental pattern length of STR) exhibit the lowest rates since the variations appearing within translated proteins might lead to different protein structures and induce deleterious effects on protein function. The longest variation type of STR among the 7 human genomes is the di-nucleotide STR motif, which occurs within the intron regions of chromosome 6. Statistics for all chromosomes with different quality settings may be downloaded directly from the developed web site.

To comprehensively display the identified polymorphic STRs and provide detailed information on selected genes, the system has a look-up table. In this table, users can easily find detailed descriptions of the selected gene and identified STRs. This web page includes Ensembl gene ID, gene name, pattern(s) of polymorphic STRs, transcript ID(s), and STR locations within the corresponding chromosome. In addition, the system also provides sequence files for two assembled families and reference sequences from Ensembl. Because of alternative splicing mechanisms in genomes, genetic regions of identified STR patterns might be affected and result in different conclusions for different transcripts. To observe all possible scenarios, the system presents all polymorphic STRs according to transcript ID. Users can click on any transcript ID and the identified results are immediately shown on the web page. To rapidly identify polymorphic STR patterns, users can click on a detected polymorphic pattern within the gene information table to display a corresponding message that is annotated with the locations framed in red. To display global sequence alignments of the identified STRs among the 7 individuals, clicking on the identified STR pattern or "Alignment Result" automatically displays the alignment results. Through these alignment procedures, users can verify and understand the polymorphic distribution of STRs among sample genomes. The multiple sequence alignments are generated in the system by ClustalW [32].

## Conclusions

In this study, an automated workflow for discovering STR polymorphisms from individual NGS sequencing data was proposed and the developed system is freely available at http://isp.cs.ntou.edu.tw/. The proposed algorithms started with performing reference mapping or *de novo *assembly of the imported NGS sequences, and the coordinate calibration was defined by mapping onto the Ensembl reference human genome. An integrated STR template profile was initially created to overcome the insertions and deletions that occurred in the reference genome or other target genomes. All possible polymorphic STR patterns could be detected automatically and precisely according to the aligned coordinate system. In this paper, polymorphic STRs from several different gene sets were applied to demonstrate the proof-of-concept, including the gene set selected by CODIS, the disease-related gene set caused by STR variations, the cross-species homologous gene set, and a human unique gene set as our evaluation datasets. In addition, all STR polymorphisms that were found within the 1000 Genomes Trio Project (6 genomes) were comprehensively identified and downloadable from the designed website. We also performed statistical analyses on both polymorphic and conserved STRs in each chromosome (except the Y chromosome), and occurrence frequencies for polymorphic STR variations between cross-species homologous genes and human-unique genes were compared for investigating the relationships between functional features or identifiable features of STR biomarkers. Therefore, STR variation frequencies for human-unique genes were clearly higher than those for cross-species conserved homologous genes, despite both gene sets exhibiting similar STR distributions and densities. This result provides an important implication in that mutations of STR elements tend not to appear within highly conserved genes among different organisms during evolutionary processes, and these cross-species conserved STRs could be considered more functionally related STRs. In other words, the polymorphic STRs that appeared within human-unique genes could be regarded as good candidates for identifiable biometric features. Focusing on the selected 477 polymorphic STRs from human-unique genes, three different categories were logically analyzed and suggested according to the 7 human genomes (considered as 3 different family groups and 7 individuals). Interestingly, we found some STR variation characteristics from human-unique genes possessing distinguishable features that could support CODIS STR verification. Furthermore, from genome-wide analysis and selection, we found a set of 26 polymorphic STRs retrieved from human-unique genes that displayed relatively higher distinguishability compared to other identified STRs. In order to understand the distributions of polymorphic STRs within each chromosome (except the Y chromosome), we compared densities of polymorphic STRs within each chromosome, and the results show that chromosome 19 had the highest density of polymorphic STRs, while chromosome 3 had the lowest density. The developed system has shown that our proposed methods could detect any polymorphic STR markers efficiently, and the proposed method could take advantage of NGS high-throughput sequencing technology and detect polymorphic STRs without manually curated and compared works. In order to efficiently provide a clear view of query results for polymorphic STRs for each gene, we have pre-processed all genes within all chromosomes (except the Y chromosome). Users will be able to perform customized sequence comparisons online for identifying all polymorphic STRs within a specified gene. In addition, users can upload their own query sequences to compare STR variations with 7 human genomes. We believe that the developed system can facilitate research involving the detection of novel STR biomarkers and the discovery of regulatory STR elements.

## Methods

### The 1000 Genome Project

To demonstrate that the proposed method is capable of detecting STR polymorphisms from NGS data, we have downloaded NGS genomic data from the 1000 Genomes Project as benchmark datasets. The 1000 Genomes Project is an ongoing international research project, the goal of which is to provide population-scale and high-coverage sequencing data world-wide. In 2010, the project completed its first phase, which included 3 pilot projects: the Low Coverage Project for providing low-coverage, whole-genome sequencing data from 179 people; the Exon Project for providing high-coverage sequencing data from 697 people, with sequencing regions limited to exonic regions of 906 randomly selected genes; the Trio Project for supporting whole-genome, high-coverage sequencing data from two families in different populations [33]. In the Trio Project, each family comprised 3 persons: father, mother, and daughter. The high-coverage sequencing data on the whole-genome scale suggested the Trio Project as an ideal sample resource for identifying various STR polymorphisms. The 1000 Genomes Trio Project files were downloaded from the NCBI FTP site in binary alignment map (BAM) format which is a *de facto *standard format for representing reference mapping results [34]. Because the files were retrieved from NCBI and mapped to the standard human genome sequences, the first step in our proposed method could be omitted. Instead, we applied SAM tools to transform the binary-archived BAM format into the plain-text SAM format, and applied the mpileup tool, which was bundled with the SAM tools to generate the consensus sequences for each individual in the Trio Project.

### Ensembl Dataset

The human genome sequences of GRCh37 from the Ensembl FTP site were also downloaded as references, and Ensembl gene annotations from BioMart interfaces were retrieved to verify genetic locations of STR motifs [35]. In the developed system, the Ensembl human genome database from version 73 was applied for analysis. A total of 56235 genes were annotated, analyzed, and compared in this study.

### CODIS markers

To verify the proposed method using previously published polymorphic STR motifs, we applied the well-known STR markers from the combined DNA index system (CODIS). The CODIS is a criminal forensic DNA database constructed by the U.S. Federal Bureau of Investigation (FBI). There were 13 highly polymorphic STR markers listed in the CODIS [5]. Each defined polymorphic STR marker within the collected 7 individual human genomes was retrieved and compared at different levels of specificity.

### Disease-related STR markers

All STR markers collected in the CODIS system were linked with neither gene regulation networks nor genetic diseases. However, several STR variations have been verified as crucial factors in causing lethal diseases, and many of the identified STRs play important roles as regulatory elements that affect gene expression. Though there are no individual medical records available for the acquired Trio samples, these verified STRs were detectable and it could be used to determine whether polymorphisms of known disease-related STR motifs occur among different individuals in the Trio Project.

### Homologous genes and human-unique genes

Quantity and quality of homologous genes provide powerful evidence for analyzing evolutionary relationships between two queried species. Investigation of STR conservation across different species has facilitated the discovery of functional STR motifs. Hence, we simultaneously collected well-defined homologous genes belonging to human, cow, dog, zebrafish, stickleback, macaque, mouse, medaka, tetraodon, and fugu as one of our experimental datasets. Through sequence alignment analysis and annotations from Ensembl, a total of 225 genes exhibiting orthologous relationships were collected, and these genes were applied to the analysis of STR polymorphisms within 7 human genomes. In contrast with the homologous gene analysis, we also collected human-unique genes by comparing all possible homologous relationships between human and the closest chimpanzee genomes. A total of 492 human-unique genes were collected for performing polymorphism analysis in this study. Polymorphic characteristics of identified STRs from human-unique genes among 7 different human genomes were considered potential candidates for STR biomarkers. To ensure the uniqueness of the collected genes, we further verified five mammalian species including gorilla, chimpanzee, macaque, orangutan, and mouse.

### System Flowchart

**Figure 6:**
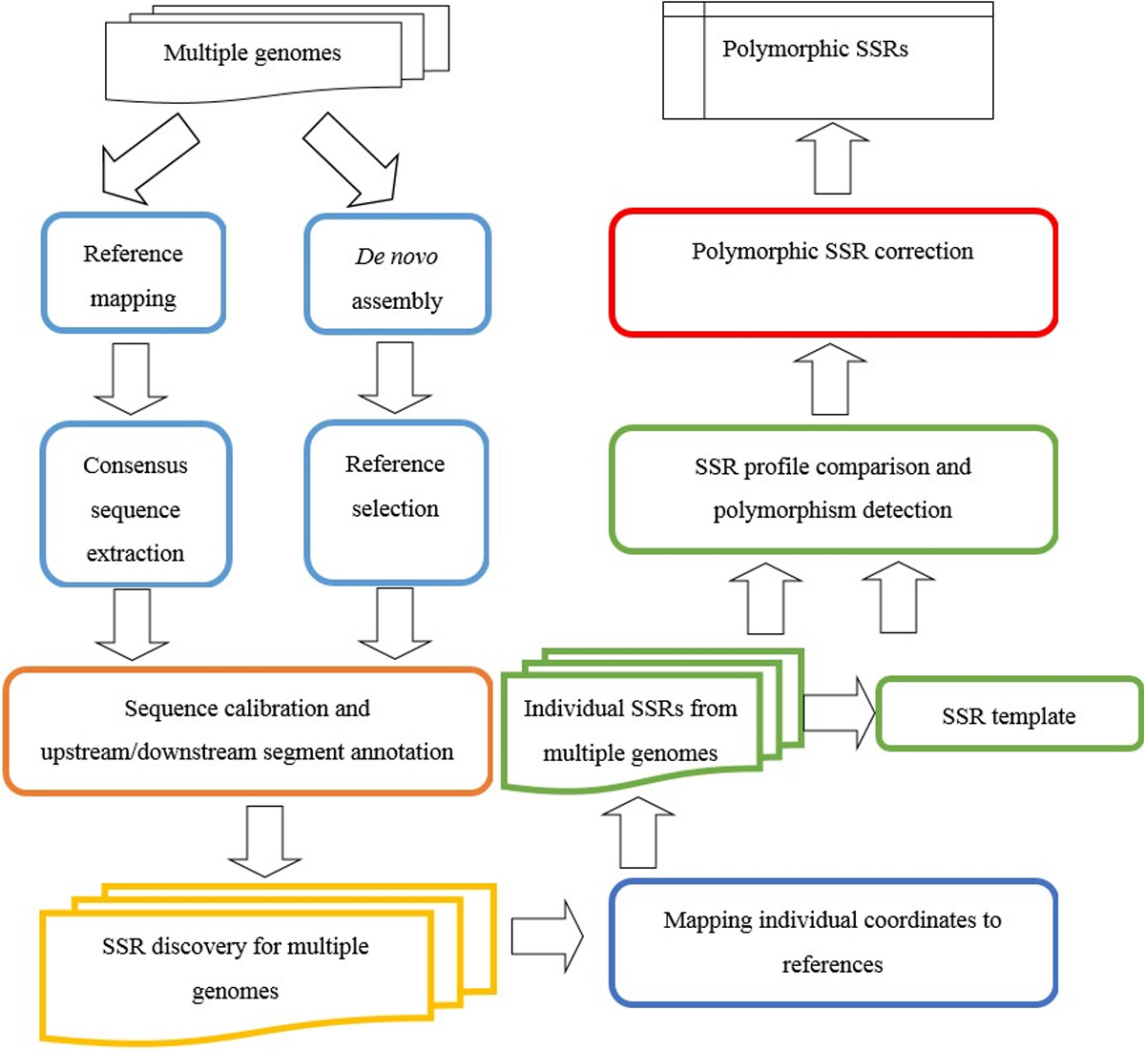
**Configuration of the proposed online system**. There are 6 major steps (represented in 6 different colors) in the designed workflow including the assembling of sequencing reads, sequence calibration, STR pattern extraction, mapping with the target reference, STR template profile construction, STR polymorphism detection, and merging neighboring STR segments.

An overview of the configuration of the proposed method is shown in Figure 6. Initially, the sequenced NGS genome datasets from different individual samples were provided as input data. There are 6 major steps that were designed for automated detection of polymorphic STRs. (1) Short reads were converted into consensus sequences in order to reduce computational complexity. There were two different standard processes for assembling NGS raw reads including reference mapping and *de novo *assembly approaches. Either approach or a combination of the two methods could be applied, depending on the target species for referencing sequences. (2) After extracting the consensus sequences from assembled contigs, each individual sequence was bias corrected, and its corresponding upstream and downstream segments were extracted. (3) Traditional *in silico *STR detection was performed on both reference and target individual consensus sequences to generate individual STR profiles. (4) Each individual sequence was aligned to a selected reference sequence in order to recognize and calibrate all corresponding locations of STR candidates. (5) Once a unified STR template profile could be constructed according to all previous STR profiles generated from imported NGS datasets, all potential polymorphic STRs were identified by automatically comparing the defined STR template profile against each individual target STR profile. (6) At the final step, a checking procedure was performed for evaluating overlapped and/or mis-recognized cases during STR retrieval processes under various parameter settings. The system was designed to include these overlapped candidates according to defined genetic locations and adjust the settings of retrieving modules (CGSSR [12]) in order to mine all possible STR patterns. The processes for each system module are described in further detail in the following sections.

### Extract consensus sequence and reference assignment

The NGS datasets are usually composed of a large amount of short reads accompanied by information regarding sequence quality. The length of short reads were usually formed from tens to hundreds of base pairs depending on various NGS machines and protocols. Since the exact location of each read is unknown, assembly processes to reconstruct the correct gene sequences from these segmented short reads were essential steps prior to genomic analysis. There are two main types of reconstruction methods available under different circumstances. If the genome of the query organism has been sequenced and published previously, a *reference mapping *approach can be applied to assemble the sequence reads. Short reads are aligned to known reference sequences, and differences between reference sequences and query reads are annotated. This approach is usually applied to sequencing model species and medically related studies. On the other hand, if no reliable reference genome is available for the target organism, a *de novo *assembly approach should be applied to the sequenced short reads. A *de novo *assembly algorithm reconstructs the original sequencing reads using read contents only, which usually requires more computational resources. Many tools are publicly available for both reference mapping and *de novo *assembly [36].

At the first stage of our proposed workflow, sequence reconstruction was completed in a manner that depended on the origin of a specific sequence. The intermediate output at this stage was consensus sequences in the standard FASTA format that were extracted from mapped results or obtained from the *de novo *assembly tools. After extraction of a consensus sequence, a reference sequence was assigned as the central representation prior to subsequent mapping processes. For the assembled results obtained from reference mapping, the reference sequences could be automatically applied. However, the output results from *de novo *assembly, i.e., the reference sequences, were picked from the individual sequencing results. A standard quality indicator such as N50 or average contig lengths could be applied for the reference selection in general. In order to compare upstream and downstream regions of target genes, we additionally collected 7500 bp from either side of each gene.

### Sequence calibration and upstream/downstream segment annotation

Although the gene sequences among the different individuals were highly similar to each other, coordinates of assembled sequences could not be directly applied across various sequencing datasets. This issue was mainly due to that random insertions or deletions caused by evolution, mutations, or reconstruction errors occurred during genomic sequence analysis. Hence, we employed NCBI BLAST+ programs to perform quick searches to further identify vague locations of the target samples [37]. Our purpose in this module was to align and correctly define both upstream and downstream segments of 7,000 bp in length for each assembled sequence. Two extra segments of 500 bp at both ends of the head of upstream and the tail of downstream segments were extended in the reference sequence to serve as key anchors for matching with all query assembled sequences and to calibrate sequence biases. After the preceding calibration processes, the extended segments with 500 bp located at both ends of the upstream and downstream regions were simultaneously discarded for all sequences. Therefore, each query sequence should contain the aligned upstream and downstream flanking segments on both sides.

### STR discovery

There are several different tools available for retrieving *in silico *STRs [11]. The ideal tools for detecting polymorphic STR markers should support STR detection while allowing different tolerance types including insertion, deletion, and substitution. In this study, we adopted CGSSR as the STR retrieving tool. CGSSR is an STR discovery tool that was developed based on autocorrelation analysis, and the kernel algorithm supports all three different types of tolerance [12]. STR motifs retrieved from each individual sequence could be mapped to the coordinates on the reference by featuring globally aligned results that were generated in subsequent steps. In this study, the obtained STRs from CGSSR were set with two tolerance rates of 90% (imperfect) and 100% (perfect), and a minimum repetition length of 20 bp.

### Mapping individual coordinates to the reference

For the problem of varied gene lengths mentioned in the previous section, sequence locations might appear as deviations within an STR profile. This location bias may lead to failed results in template-building procedures; thus, all corresponding STR segments among different individuals should be identified through an appropriate approach. Each sequence was therefore calibrated in advance regarding their system coordinates comparing to the assigned reference sequence through a global pairwise alignment. In this study, we applied the EMBOSS stretcher program to perform global alignment between the reference sequence and each individual target sequence. The aligned results were then employed as the data resource for coordinate transformation [38]. Each discovered STR record within an individual sequence was annotated with the information for corresponding locations in the reference gene sequence, repeat motif pattern, and repeat times. The collection of all mapped-coordinate STR records was finally defined as an STR template profile.

### STR template and polymorphic STR construction

Since an STR profile contained all retrieved STR motifs from an individual genome under an identical coordinate system, the STR polymorphism could then be observed by comparing with all the remaining STR profiles. To efficiently and effectively list all polymorphic STR candidates, a representative and comprehensive STR template was built by union operations from the reference profile and all other individual profiles. It should be noted that all STR patterns were compared under rotational tolerance because the basic STR pattern might be shifted as a result of point mutations or insertion/deletion polymorphisms. Once the template profile was constructed, polymorphic STRs could be identified easily by comparing all STR records within the accumulated template profile against each individual STR profile. Since all coordinates of STRs were aligned to the reference sequence, the known gene annotations from the reference gene could be applied to each individual STR motif for assigning appropriate genetic location information. After constructing a comprehensive and annotated STR profile for each individual, we only have to judge the existence and the repeat number of a specific STR pattern at a corresponding location, and therefore the system could respond to a query in real time and verify all polymorphic STRs.

### Merging neighboring STR segments

Due to mutations and gap noises that appear within a repeat DNA sequence, polymorphic STRs could be erroneously divided into several segments. This situation caused statistical errors during cross-sample comparison. In order to avoid such errors, the system provides a merging function for neighboring segmented patterns according to their patterns and overlapped conditions. The merging module could reunite disconnected STR segments into one motif according to previously defined coordinate information. Another potential problem is N/A nucleotides; these require adjusting one of the parameters in CGSSR to find shorter STR patterns that might not have been found in previous steps. Through this proposed mechanism, a comprehensive STR profile for each gene could be successfully constructed.

### Function of comparing customized DNA sequences

To design an integrated system for customized services, the system provides users the ability to upload their own gene sequences and discover all polymorphic STRs against the benchmark human genomes. Once a customized sequence is uploaded, the designed system automatically blasts the query gene sequence against these genomes to identify its corresponding gene. The query sequence is then scanned to detect all STR motifs, and their corresponding STR profiles will be created according to previously introduced modules. The online system is freely available at http://isp.cs.ntou.edu.tw/.

### Additional material

Additional file 1: Supplementary Document 1. A table of 477 polymorphic STR patterns retrieved from 492 human-unique genes. All related genetic information for each identified STR is described in detail.

Additional file 2: Supplementary Document 2. A table of 26 STRs selected from 477 polymorphic STRs based on specific conditions. All STRs were clustered into three different groups according to individual, family, and ethnic relationships.

## List of abbreviations

STR - Short Tandem Repeat

pSTR - Polymorphic STR

TLgene - Total length of gene

TLssr - Total length of SSR

TNgene - Total number of gene

TNpssr - Total number of polymorphism SSR

TNssr - Total number of SSR

UTR - Untranslated Region

Chr/Chrom - Chromosome

BLAST - Basic Local Alignment Search Tool

Mbp - Mega base pairs

NCBI - National Center for Biotechnology Information

ENBL - Ensembl

CODIS - Combined DNA Index System

NGS - Next Generation Sequencing

BAM - Binary Alignment Map

SAM - Sequence Alignment Map

## Competing interests

The authors declare that they have no competing interests.

## Authors' contributions

CMC, CPS and TWP conceived the algorithms. CMC, CPS, and YLL implemented the algorithms, performed the experiments. CMC and CPS wrote the manuscript. TWP, HTC, and CHH evaluated the systems, and proofread and revised the manuscript. All authors read and approved the final manuscript.

## Supplementary Material

Additional file 1 **Supplementary Document I: A total of 477 polymorphism STR patterns retrieved from 492 human-unique genes**. Sorted by Avg. Dev. and Max. Dev.; Chrom is an abbreviation of chromosome; Enbl represents Ensembl; NA12878 represents CEU child, NA12891 for CEU father, and NA12892 for CEU mother; NA19238 represents YRI mother, NA19239 for YRI father, and NA19240 for YRI child. Region: 1=Intron, 2=Upstream, 3=Downstream, 4 = 5'UTR, 5=Coding, 6 = 3'UTR, 7=Undefined exon. Click here for file

Additional files 2 **Supplementary Document II: A total of 26 STRs selected from 477 polymorphic STRs based on specific conditions**. All STRs were sorted by different levets of family (Type I), individual (Type II), and ethnic (Type III). Chrom is an abbreviation of chromosome; Enbl represents Ensembl; NA12878 represents the CEU child, NA12891 the CEU father, and NA12892 the CEU mother; NA19238 represents the YRI mother, NA19239 the YRI father, and NA19240 the YRI child. The symbol of "*" in the GID column represents genetic variation of single nucleotide polymorphism (SNP). Click here for file

## Declarations

The publication charges of this article were funded by the Ministry of Science and Technology, Taiwan (MOST 103-2627-B-019 -003 and MOST 103-2221-E-019 -037 to Tun-Wen Pai).

This article has been published as part of *BMC Genomics *Volume 15 Supplement 10, 2014: Proceedings of the 25th International Conference on Genome Informatics (GIW/ISCB-Asia): Genomics. The full contents of the supplement are available online at http://www.biomedcentral.com/bmcgenomics/supplements/15/S10.
